# Virtual reality as an engaging and enjoyable method for delivering emergency clinical simulation training: a prospective, interventional study of medical undergraduates

**DOI:** 10.1186/s12916-024-03433-9

**Published:** 2024-06-03

**Authors:** Risheka Walls, Priyanka Nageswaran, Adrian Cowell, Tunav Sehgal, Thomas White, James McVeigh, Stefan Staykov, Paul Basett, Daniel Mitelpunkt, Amir H. Sam

**Affiliations:** 1 Imperial College School of Medicine, St Dunstan’s Rd, Charing Cross Campus, London, W6 8RP UK; 2 Imperial College Faculty of Medicine Digital Education Office, Exhibition Road, South Kensington, London, SW7 2BX UK; 3 Imperial College Digital Media Lab, Exhibition Rd, South Kensington, London, SW7 2BX UK; 4 Stats Consultancy, Amersham, UK

**Keywords:** Virtual reality, Simulation, Innovation, Medical emergencies

## Abstract

**Background:**

It is a requirement that medical students are educated in emergencies and feel well prepared for practice as a doctor, yet national surveys show that many students feel underprepared. Virtual reality (VR), combined with 360-degree filming, provides an immersive, realistic, and interactive simulation experience. Unlike conventional in-person simulation, it is scalable with reduced workforce demands. We sought to compare students’ engagement and enjoyment of VR simulation to desktop computer-based simulation.

**Methods:**

We conducted a prospective, interventional, evaluation study. The study was carried out on final year medical students undertaking their Pre-Foundation Assistantship (*n* = 116) at Imperial College School of Medicine (ICSM) in London. We compared objective engagement, subjective engagement, and subjective enjoyment of VR simulation to desktop computer-based simulation using cardiac arrest and life-threatening asthma scenarios. Engagement was measured objectively using students’ physiological parameters, including heart rate and eye tracking, and facilitator observations using the validated ‘Behavioural Engagement Related to Instruction’ (BERI) protocol. Students’ subjective engagement and enjoyment levels were measured using a post-session survey.

**Results:**

Students’ maximum heart rates were significantly higher during VR simulation with a mean difference of 4.2 beats per minute (3.2 to 5.2, *p* < 0.001), and eye tracking showed they spent a significantly greater mean percentage of time of 6.4% (5.1 to 7.7, *p* < 0.001) focusing on the scenarios in VR compared to standard desktop. Qualitative data showed students enjoyed and felt engaged with the sessions, which provided a safe space for learning.

**Conclusions:**

Our study shows that students found VR simulations enjoyable and were more engaged compared to standard desktop simulation. This suggests that 360-degree VR simulation experiences provide students with immersive, realistic training, which is scalable, giving them the unique opportunity to manage emergencies and work within emergency teams, which would not typically occur during traditional training.

**Supplementary Information:**

The online version contains supplementary material available at 10.1186/s12916-024-03433-9.

## Background

Attrition of doctors, rota gaps and industrial action within the National Health Service are becoming increasingly common. Training more doctors is a frequently cited solution, yet the current learning environment is not geared for such capacity changes, and many existing medical students already feel unprepared for the technical and non-technical aspects of practice. Simulation-based medical education (SMBE) describes artificially reproducing authentic medical scenarios to accomplish educational outcomes. SBME utilises experiential learning to guide learners and is a teaching style that is commonly used in the medical curricula to effectively complement patient safety [1]. Complex, high-stakes emergency scenarios are often practiced and assessed in SBME to build self-confidence in learners during these demanding settings without any real consequence to patient safety [2]. In medicine, SMBE is typically delivered through in-person simulation, an effective but resource intensive teaching method [3], which is used sparingly due to timetabling and workforce pressures. Comparable industries, such as aviation, use highly sophisticated simulators to train pilots in a realistic and immersive manner. However, this type of simulation is expensive and therefore inaccessible in the healthcare setting. Computer-based simulation on a desktop is a seemingly inexpensive and accessible resource given most students have access to a computer personally or through institutions. It however lacks elements of immersion and engagement. Over the past two decades, SBME has advanced with evolving technology and more recently seen an adoption of virtual reality (VR) as a technique of technology-enhanced learning [4]. VR simulation combined with 360-degree filming may provide a sustainable option for simulation training. Learners can safely be immersed in, interact with and influence a realistic clinical setting, whilst also receiving core teaching [5, 6]. VR provides an opportunity for blended learning with existing in-person clinical placements and addresses the gaps in clinical exposure in a cost-effective and scalable manner. VR resources can be readily shared across healthcare sectors and educational establishments.

VR describes a human–computer interface that provides users with an immersive computer simulated environment. Immersion requires a high level of attention and describes being engaged in the environment to the point where all the focus is on that particular task and there is a strong feeling of being there [7]. This element is key in medical training where realism, rather than gamification, is valued and total immersion tests students’ ability to remain level headed in dynamic scenarios. VR is not new technology, with the term first being described by Jaron Lenier in the mid-1980s [8]. Over time, and with advancements in technology, VR has grown to be used commercially and its technology is well established in gaming. Studies have found that immersion with VR through experiential learning improves learning [9, 10]. Using VR has been shown to be more useful for education, and learners have subjectively found its use exciting or interesting [10, 11]. Learning with simulations has shown to be an effective teaching method for knowledge retention and overall student performance [12–14]. Higher levels of student engagement result in higher levels of academic achievement [15, 16]. We combined the two key features of simulation, realism and immersion, through 360-degree filming of a ‘real life’ emergency delivered interactively in VR to train clinical year medical students in managing cardiac arrest and life-threatening asthma.

Student engagement with teaching can be measured objectively and subjectively. Subjective methods include self-reported levels of engagement using engagement assessment tools [17, 18]. Objective methods of student engagement include monitoring physiological variables such as heart rate (HR) or eye movements [19–21] and validated observer tools of engagement such as the Behavioural Engagement Related to Instruction (BERI) protocol [22]. Heart rate levels are an effective monitor of student engagement levels with an increase in heart rate seen during periods of involvement or interaction [19, 20]. Eye movement information can provide valuable insight into engagement and attention levels. Movements can be defined as saccades, rapid eye movements from point-to-point at high velocities (> 300°/s) or fixations, low-velocity eye movements (< 100°/s), which indicate lingering of the gaze [23]. Overall, longer fixations imply greater cognitive processing and regular saccades typically indicate distraction from a task [24, 25]. Fixations can also take place within areas-of-interest (AOI), which are pre-determined target regions that subjects should be placing attention to and longer periods of eye movements within the AOIs indicate higher engagement [23, 26]. In this study, we sought to understand whether students found VR simulations engaging and enjoyable and how this compared to desktop computer-based simulation.

## Methods

The Imperial College London Education Ethics Review Process (study ref. EERP2122-038) approved the study on 24 January 2022. We conducted a prospective interventional evaluation study of final year medical students (*n* = 116) from 27 April 2022 to 22 June 2022. The study was carried out at Imperial College School of Medicine (ICSM) in London. All final year medical students undertaking their Pre-Foundation Assistantship (PFA) at ICSM were screened for eligibility. Participants gave informed consent before taking part. All those who attended the simulations agreed to partake in the research, and it was clearly highlighted to them beforehand that those who chose not to participate in the research would still be given the same learning opportunities. Our inclusion criteria included students who were able to give informed consent. The exclusion criteria for eye tracking data included students who had undergone any eye surgery or students wearing bifocal/trifocal glasses for the study. Exclusion criteria for the heart rate data included participants with any pre-existing cardiac rhythm abnormalities. These conditions were excluded as they interfere with the eye tracking and the heart rate monitoring leading to inaccurate data. Students were provided with written information sheets and a verbal introduction with question-and-answer opportunities. The purpose of the study proposed to students was to understand whether VR simulation is an engaging and enjoyable form of simulation that allows them to experience emergency scenarios that they might not see in real life. Students were able to withdraw their information within 2 weeks.

**Fig. 1:**
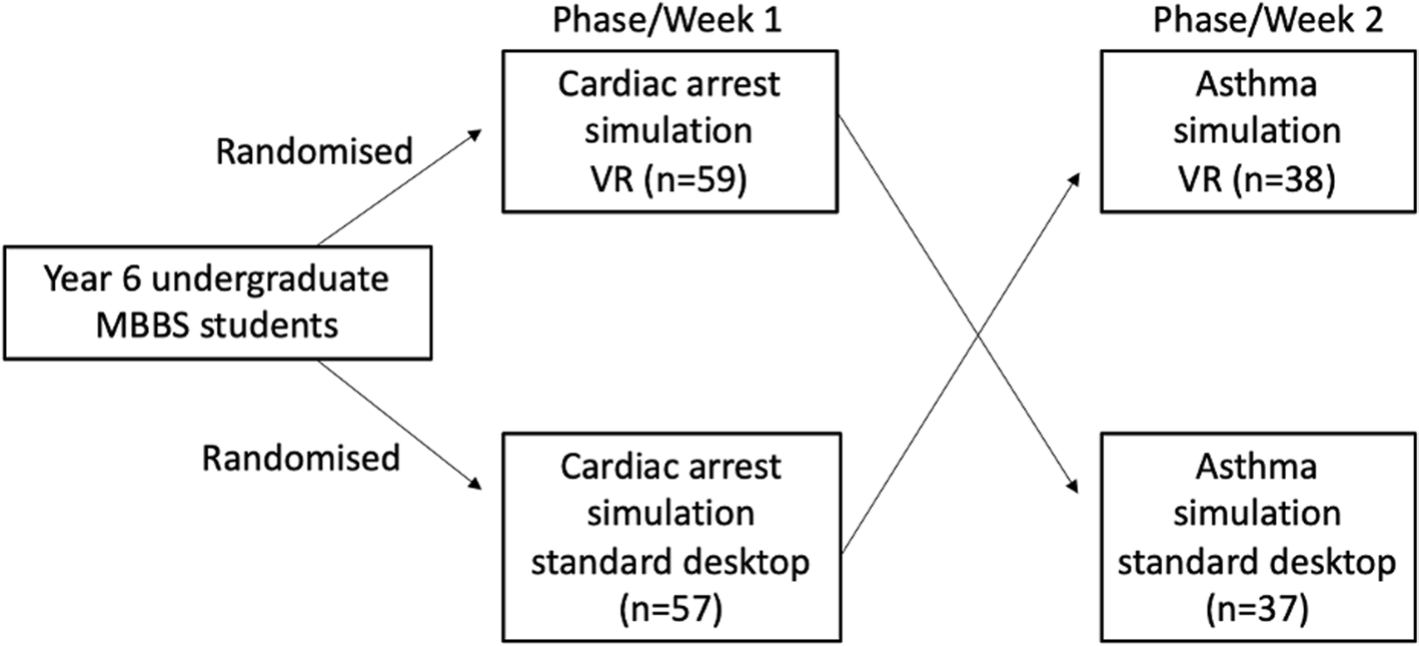
Distribution of students into scenario type and delivery method sub-groups

The study aimed to compare student engagement and enjoyment levels between VR simulation and desktop computer-based simulation during two simulation experiences of a cardiac arrest and life-threatening asthma attack (see Additional File 1: Images S1 and S2). Both scenarios were 15 min and created using 360-degree filming of acted-out scenarios. Interactivity was introduced with questions and variable scene branching dictated by student responses. The format of the 20-min debriefs for both groups were the same, consisting of discussions around the technical and clinical skills involved in managing both scenarios and the non-technical skills involved. All students experienced both scenarios, and all students experienced VR once and standard desktop once. Students were split into groups of 10–15 for each of the VR group and the desktop group. Students did not know which scenario they would be presented with first. In the first phase of the study, students were randomly assigned to either experience cardiac arrest in VR or on standard desktop. In the second phase of the study, those who were previously in the VR group for cardiac arrest transitioned to life-threatening asthma on standard desktop, and those previously in the cardiac arrest standard desktop group transitioned to life-threatening asthma in VR. One hundred ninety-one datasets were collected in total (see Fig. 1).

Engagement was measured objectively using students’ physiological parameters (heart rate and eye tracking) and facilitator observations using the validated BERI protocol [22]. Heart rates were monitored using the Polar H10 heart rate sensor. Eye tracking data was obtained from Tobii VR4 platform with spotlight technology built into the Pico Neo 3 Pro Eye VR headsets. Eye tracking parameters measured included pupillary saccades and fixations within assigned areas of interest. Students’ subjective engagement and enjoyment levels were measured using a post-session survey which was collected immediately after the session using an online questionnaire and included a combination of qualitative and quantitative data.

Each of the simulations consisted of a baseline period, followed by alternating scenes and questions. Data on the students’ heart rate and whether they were on task (measured using eye tracking) were recorded serially over the simulations. For each student, the data was reduced to a single measurement per section of the simulation (either the baseline period, or individual questions and scenes). For heart rate monitoring, both the mean and maximum value during the section was calculated. For engagement, the percentage of measurements where the student was on-task was measured using eye tracking.

The analyses of both the VR data and the desktop data used equivalent methods. Due to the multiple measurements for each student over time, the analyses were performed using mixed (multilevel) linear regression models. The outcome variable was the heart rate/on task measurement for each section of the simulation (either scene or question). The equivalent outcome at baseline was used as covariate in the analyses. Also included as a covariate was the section number, to allow for trends in outcome over the course of the simulation. Two level regression models were used with individual measurements for each section nested within students. An autoregressive structure for the residuals was used, which allows each measurement to be correlated with the preceding measurement.

The first analysis of the joint VR and standard desktop data compared the difference between techniques for the ‘baseline’ measurements made before the main part of the simulation. One measurement per student was included in these analyses, with the unpaired *t*-test used for the comparisons. The regression analyses were adjusted for differences during the baseline period as there were differences during the baseline period.

The analysis of the main simulation outcomes was performed using similar statistical methods to those previously described. Initially, the difference between scenes and questions was examined for all scenarios combined. Subsequently, the interaction between technique (standard desktop or VR) and scenario (scenes or questions) was included. If the interaction was significant, this would suggest that the difference techniques varied for scenes and questions. Where a significant interaction was present, the technique differences were quantified for each scenario. If the interaction was not significant, it was omitted from the analysis, and a single difference between techniques was quantified.

## Results

### Engagement levels using physiological parameters

The heart rate values during the baseline period were significantly higher for the VR technique. This was adjusted for when analysing the main part of the simulation using regression analyses (see Additional File 2: Table S1). The results presented in Table 1 summarise the differences in outcome between standard desktop and VR methods. Summaries for each method are reported, along with a mean difference and corresponding confidence interval. As there were differences during the baseline period (see Additional File 2: Table S1), the adjusted differences presented in Table 1 are notably lower than the raw difference in outcome between groups. However, the fact that the differences were statistically significant implies that there were differences in outcome between techniques even after allowing for the baseline differences.

**Table 1:** Comparison of heart rate outcomes between standard desktop and VR groups after adjusting for differences during the baseline period using regression analyses

Outcome	Simulation	Data summaries—standard desktop ^(+)^		Data summaries —VR ^(+)^		Difference ^(*)^	*p*-value
		*N*. students	Mean ± SD	*N*. students	Mean ± SD	Mean (95% CI)	
Mean heart rate (bpm)	Asthma	34	83.9 ± 11.8	29	93.9 ± 15.5	2.9 (1.9. 3.9)	**< 0.001**
	Cardiac arrest	45	78.8 ± 10.9	25	94.1 ± 12.0	2.9 (1.4, 4.4)	**< 0.001**
	Both	79	81.5 ± 11.7	54	94.0 ± 14.3	3.0 (2.2, 3.8)	**< 0.001**
Maximum heart rate (bpm)	Asthma	34	88.5 ± 13.2	29	98.4 ± 16.2	4.8 (3.4, 6.1)	**< 0.001**
	Cardiac arrest	45	81.9 ± 12.2	25	97.8 ± 12.9	2.6 (0.7, 4.4)	**0.008**
	Both	79	85.4 ± 13.1	54	98.2 ± 15.1	4.2 (3.2, 5.2)	**< 0.001**
% time on task	Asthma	36	86.1 ± 16.3	38	99.0 ± 5.4	7.9 (5.7, 10.2)	**< 0.001**
	Cardiac arrest	57	89.7 ± 13.4	57	99.4 ± 2.5	5.3 (3.7, 6.9)	**< 0.001**
	Both	93	88.0 ± 15.0	95	99.2 ± 4.2	6.4 (5.1, 7.7)	**< 0.001**

( +) Summaries based on one observation per section of the simulation per student

(*) Difference from mixed regression models analysis. Reported as outcome for VR minus outcome for standard desktop

The mean of the mean heart rates was significantly higher during VR compared to desktop for both simulations combined, by 3.0 bpm (2.2 to 3.8, *p* < 0.001), after adjusting for baseline differences. The mean of the maximum heart rates was also significantly higher during VR compared to desktop for both simulations combined, by 4.2 bpm (3.2 to 5.2, *p* < 0.001), again, after adjusting for baseline differences. For time on task measured using eye tracking, the percentage of time on task was 6.4% higher (5.1 to 7.7, *p* < 0.001) for the VR technique than for standard desktop.

### Observed engagement levels

When analysing the observed engagement levels of the 191 student experiences, students were engaged for a mean of 93.61% (SD = 0.048) of time when using VR, compared to 88.13% (SD = 0.087) of time for students using standard desktop. An independent samples *T*-test with a 95% confidence interval showed a statistically significant difference in observed engagement between the VR group and the desktop group (*p* = 0.001).

### Subjective engagement and enjoyment levels

A total of 191 feedback datasets were collected on students’ subjective engagement and enjoyment levels using a five-point Likert-style questionnaire. A Pearson chi-square test was performed to determine whether the simulation scenario (cardiac arrest or life-threatening asthma) had any significant impact on engagement, and enjoyment levels gave a *p*-value of 0.265 and 0.494 respectively. We therefore concluded that there was no significant difference between the simulation scenarios with regard to the subjective engagement and enjoyment levels. The results of the simulations were then pooled to create two distinct groups: VR and standard desktop (Table 2). The average engagement level was 4.25 when using a standard desktop (SD = 0.88), compared to 4.48 when using VR (SD = 0.75). The average enjoyment level was 4.05 when using a standard desktop (SD = 0.96), compared to 4.44 using VR (SD = 0.76). A Pearson chi-square test was performed to determine whether the type of simulation (VR or standard desktop) had any significant impact on engagement, and enjoyment levels gave a *p*-value of 0.076 and 0.009, respectively.

**Table 2:** Comparison of subjective engagement and enjoyment levels between standard desktop and VR groups for both cardiac arrest and asthma scenarios combined

Outcome	Standard desktop		VR		*p*-value
	*N*. students	Mean ± SD	*N*. students	Mean ± SD	
Subjective engagement (1–5)	94	4.25 ± 0.88	97	4.48 ± 0.75	**0.076**
Subjective enjoyment (1–5)	94	4.05 ± 0.96	97	4.44 ± 0.76	**0.009**

## Discussion

It is undoubted that student engagement is important in education. An engaged student is likely to learn more and retain more [27]. Heart rate and engagement are closely correlated [28]. Superior learning performance [29], greater cognitive effort, and higher order problem solving are all correlated with an increased heart rate [30, 31]. Our results suggest a significant difference in mean and maximum heart rates during VR simulation compared to standard desktop simulation. Literature suggests that the heart rate change signifying engagement is typically around 5 bpm, which is mirrored in our study where the difference in the maximum heart rates between standard desktop and VR is 4.2 bpm [30]. Heart rates were monitored using a heart rate monitor, which was connected to a tablet for the desktop group, and to the VR headset via Bluetooth for the VR group. Interruptions in heart rate monitoring were therefore easier to detect and rectify for the desktop group where we could see the tablet, which explains why more students had heart rate monitoring data for the desktop group.

Blended learning is an established feature of undergraduate and postgraduate medical education. Studies confirm that students who watch videos and participate in classroom teaching perform better than those who only received traditional face-to-face teaching [32]. Furthermore, dynamic video-based learning improves performance compared to static text and pictures [33]. Eye tracking provides key physiological indicators of engagement and focus*.* Fixations define a location where a student is focusing their visual attention. When an individual is focusing on an area, the assumption is that they are retrieving and processing information in that area. We defined these as areas of interest, and the percentage of time students’ gaze was fixed on that area of interest was recorded. Our results show that students spend a significantly greater percentage of time focusing on the predefined areas of interest which reflect the scene or task in front of them (‘on task’) during VR simulation when compared to standard desktop.

Technical characteristics of the desktop computer and the VR glasses can affect the quality of the content, and we took steps to mitigate against this influencing user immersion. We used screens readily accessible to students, and whilst the VR headset had a higher resolution than the desktop, there was a loss in quality due to projection of the image onto a sphere in VR. Video framerates matched in both modalities and remained below the refresh rate. Video playback of the scenarios was used in VR instead of streaming, and scenarios were streamed using an Ethernet connection on the desktops to ensure there were no latency issues. The audio was delivered through identical headphones for both VR and desktop.

Our mixed methods study using physiological parameters, objective engagement observations, and subjective engagement and enjoyment levels shows that delivering simulation in the form of VR is more enjoyable and engaging than watching a 360-degree video on a desktop. This study is the first to evaluate the engagement and enjoyment of VR in the context of undergraduate medical training in an era where there is a need to train more doctors within the existing and limited hospital facilities. We have shown that immersive VR simulation is an accessible and scalable form of teaching life-threatening emergency scenarios which are difficult to access in a clinical setting. Unlike in-person simulation, VR simulations can be delivered to 30 to 60 students simultaneously with just one or two facilitators. This compares favourably to in-person simulation which requires one to two facilitators for up to four students, often with other students watching and critiquing rather than taking part themselves. Virtual reality headset costs may be a perceived barrier to implementation. However, good VR headsets (£300) compare favourably to the cost of desktop computers and iPads and markedly less than simulation suites (£100 k to 1 million). They do not incur the repeating costs of room and timetabling spaces, facilitators, and actors, thereby providing a cost-effective and re-usable method of teaching. VR simulation is therefore a scalable opportunity to educate and train more students effectively where space, time, and money is at a premium. However, we appreciate that in-person simulation also allows training in technical skills. Whilst not available with these 360-degree VR simulations, this is currently an evolving field in VR development with advancements in haptic feedback.

Given students experienced the VR and desktop scenarios on separate occasions, there was potential for contamination between groups with students relaying their experience of one format to a group that had not yet experienced it. Students were expected to account for their own personal experience. We did not ask about prior exposure to VR technology and acknowledge that a novelty effect may have influenced the result in favour of VR. There were no reports of kinetosis, hearing loss, or anxiety, but it is important to screen for these prospectively in future studies. This study did not evaluate knowledge retention, which would be difficult to quantify in this setting as it requires re-testing of students after they qualify as doctors. This would bring in numerous additional variables including re-recruitment difficulty and variation in postgraduate exposure to emergency scenarios between students. Most simulation in medicine is in-person simulation. This study does not compare VR simulation to in-person simulation as this comparison would require external eye tracking or tracking through augmented reality devices. It would be influenced by movement around the room which affects both eye movement and heart rate, whereas students physically move very little during VR scenarios. These VR scenarios are not a replacement for in-person simulation but allow a greater number of students to participate in an immersive experience, alongside exposure to a wider breadth of emergency scenarios. We plan to compare our VR simulation to in-person simulation in the future. Given the difficulties in monitoring physiological parameters for in-person simulation, we plan to use a qualitative approach to evaluate subjective engagement and enjoyment between the two arms of the study.

## Conclusions

Our study has shown that VR simulations are enjoyable and more engaging compared to standard desktop simulation. The findings suggest that 360-degree VR simulation provides immersive and realistic training allowing students to manage emergencies that they may not experience during traditional training. VR-based learning blended with in-person clinical attachments may represent the future of health education, especially for clinical experiences which cannot be guaranteed in real life yet require situational awareness and the ability to remain level-headed in dynamic real-life scenarios.

### Supplementary Information

Additional file 1. Images S1-S2. Image S1-Screenshot of the cardiac arrest scenario, created with 360-degree filming of an acted-out scenario. Image S2-Screenshot of the life-threatening asthma scenario, created with 360-degree filming of an acted-out scenario.

Additional file 2. Table S1. Table S1-Comparison of baseline data between standard desktop and VR groups.

## Data Availability

We commit to making the relevant anonymised data available on reasonable request. All data collected will be made available immediately following publication for 36 months to researchers who provide a methodologically sound proposal. This data will be available to achieve aims in the approved proposal. All proposals should be directed to the corresponding author.

## Abbreviations

AOI: Areas of interest
BERI: Behavioural engagement related to instruction
HR: Heart rate
ICSM: Imperial College School of Medicine
PFA: Pre-Foundation Assistantship
SBME: Simulation-based medical education
VR: Virtual reality
