# Supplementary material for: Virtual reality as an engaging and enjoyable method for delivering emergency clinical simulation training: a prospective, interventional study of medical undergraduates
Source: BMC Med. 2024 Jun 3;22:222. doi: 10.1186/s12916-024-03433-9 (PMC11149210; doi:10.1186/s12916-024-03433-9)

**Additional File 1**

Image S1: Screenshot of the cardiac arrest scenario, created with 360-degree filming of an acted-out scenario.


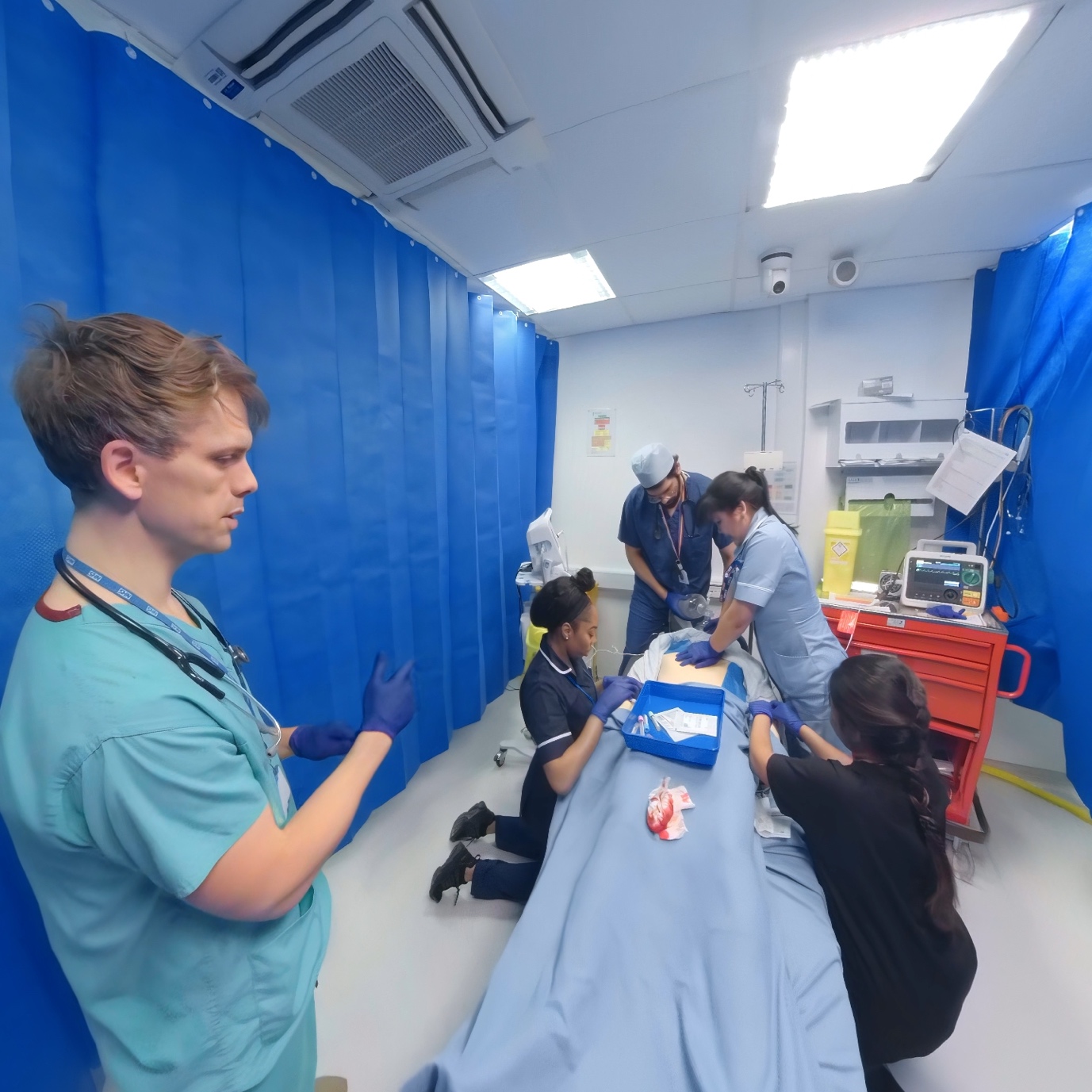


Image S2: Screenshot of the life-threatening asthma scenario, created with 360-degree filming of an acted-out scenario.


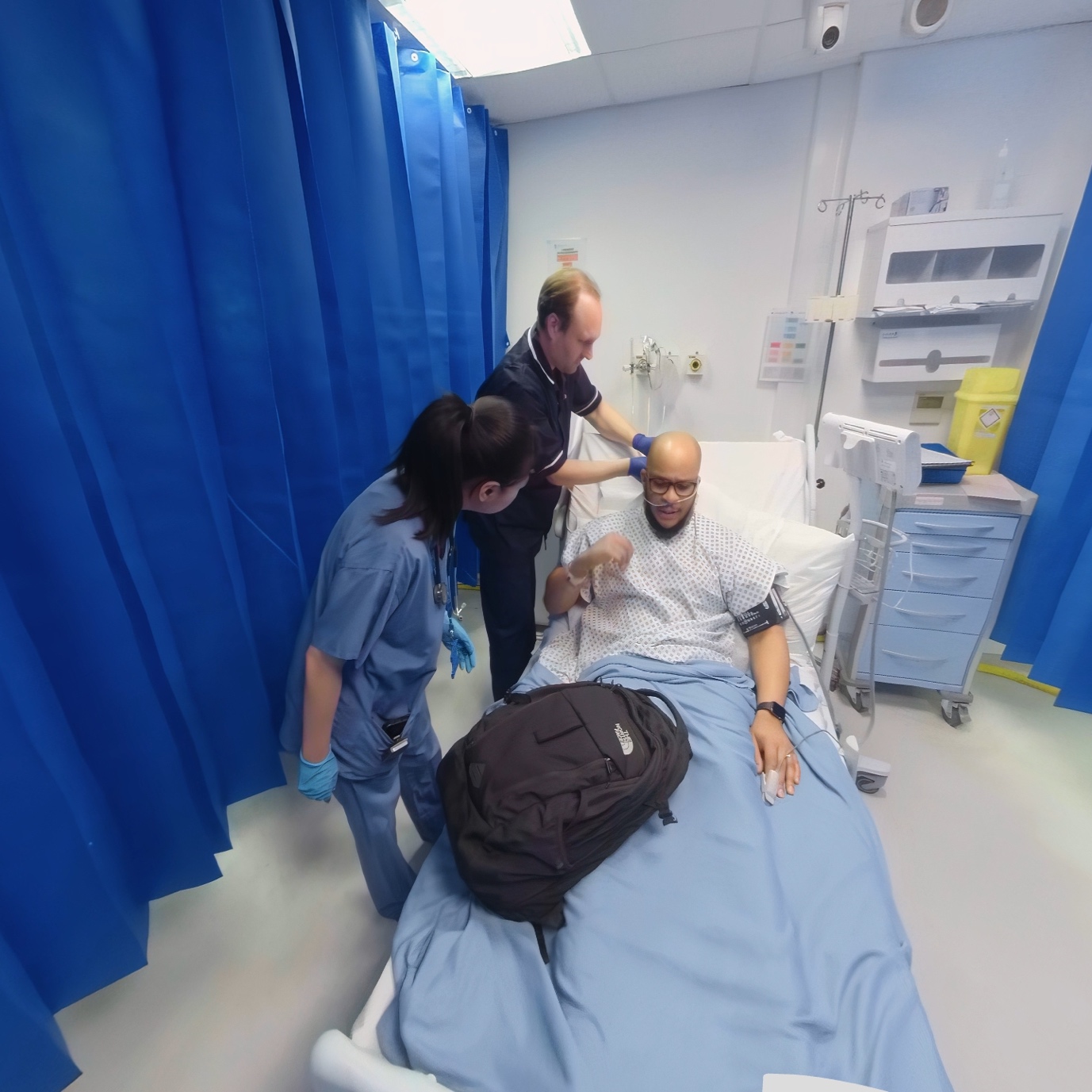

Supplement: Supplementary file 1 — Additional file 1. Images S1-S2. Image S1-Screenshot of the cardiac arrest scenario, created with 360-degree filming of an acted-out scenario. Image S2-Screenshot of the life-threatening asthma scenario, created with 360-degree filming of an acted-out scenario. [file 12916_2024_3433_MOESM1_ESM.docx]
