# Supplementary material for: Virtual reality as an engaging and enjoyable method for delivering emergency clinical simulation training: a prospective, interventional study of medical undergraduates
Source: BMC Med. 2024 Jun 3;22:222. doi: 10.1186/s12916-024-03433-9 (PMC11149210; doi:10.1186/s12916-024-03433-9)
Supplement: Supplementary file 2 — Additional file 2. Table S1. Table S1-Comparison of baseline data between standard desktop and VR groups. [file 12916_2024_3433_MOESM2_ESM.docx]

**Additional File 2**

Table S1: Comparison of baseline data between standard desktop and VR groups.

| Outcome | Simulation | Standard desktop | | VR | | P- |
| --- | --- | --- | --- | --- | --- | --- |
|  |  | N. students | Mean ± SD | N. students | Mean ± SD | value |
| Mean heart | Asthma | 34 | 84.7 ± 12.1 | 29 | 92.9 ± 16.0 | **0.02** |
| rate (bpm) | Cardiac arrest | 45 | 80.6 ± 11.8 | 25 | 94.8 ± 11.0 | **<0.001** |
|  | Both | 79 | 82.4 ± 12.0 | 54 | 93.8 ± 13.9 | **<0.001** |
|  |  |  |  |  |  |  |
| Maximum | Asthma | 34 | 93.1 ± 15.0 | 29 | 100.4 ± 16.5 | 0.07 |
| heart rate (bpm) | Cardiac arrest | 45 | 86.0 ± 13.4 | 25 | 101.8 ± 11.8 | **<0.001** |
|  | Both | 79 | 89.2 ± 14.5 | 54 | 101.1 ± 14.4 | **<0.001** |
|  |  |  |  |  |  |  |
| % time on | Asthma | 36 | 80.8 ± 7.1 | 38 | 99.6 ± 0.7 | **<0.001** |
| task | Cardiac arrest | 57 | 82.7 ± 14.8 | 57 | 99.5 ± 1.1 | **<0.001** |
|  | Both | 93 | 81.0 ± 15.0 | 95 | 99.5 ± 1.0 | **<0.001** |
